# What dose of anti-snake venom should be given in severe neuroparalytic snake bite?

**DOI:** 10.4103/1817-1737.74281

**Published:** 2011

**Authors:** Avinash Agrawal, Alok Gupta, Arjun Khanna

**Affiliations:** *Department of Internal Medicine, Chattrapati Shahuji Maharaj Medical University, Lucknow, Uttar Pradesh, India. E-mail: icuexpert@gmail.com*

Sir,

Indian cobra (*Naja naja*) and Common Indian krait (*Bungarus caeruleus*) are two important species of elapid snakes found in India and are responsible for most of the cases of neurotoxic snake bite. Respiratory failure is the most important cause of morbidity and mortality in victims of neurotoxic snake bite.[1,2] Cobratoxin and α-bungarotoxin act postsynaptically by binding to acetylcholine receptors on the motor end plate while β-bungarotoxin and crotoxin act presynaptically and prevent release of acetylcholine at the neuromuscular junction.

Timely administration of anti-snake venom (ASV) along with cardiorespiratory support is the only effective treatment available for neurotoxic snake bite.[3,4] ASV is the most effective when administered early enough to neutralize venom in the circulation before it reaches the target site. However, there is no universal consensus on the optimal dose and protocol of ASV administration. Higher doses of ASV had been used earlier with the hope of early recovery.[5] Other investigators have found no significant difference on survival outcome and duration of ventilation while comparing high dose ASV regimens with low dose ASV regimens.[6]

Fifty-eight patients with severe neurotoxic snake bite with respiratory failure were admitted to MICU during the study period. Of this there were 41 males and 17 females. The age range was 13–55 years. Mean duration of time to reach hospital was 5.7 hours. All 58 patients had symptoms of neurotoxicity, ranging from ptosis, dysphagia, respiratory distress, and generalised loss of power to alteration in the level of consciousness. All patients were in the respiratory failure as evidenced by single breath count (SBC), if possible and by ABG analysis. All patients were administered an initial bolus dose of 200 ml ASV as a continuous infusion over 2 hours followed by the repeated doses of 100 ml ASV every 6 hours until the patient showed signs of neurological recovery.

In our study, we have used a high initial bolus dose of ASV, i.e. 200 ml to all patients with respiratory failure keeping in mind that such a high dose would neutralize maximum amount of toxin circulating in the blood, which would otherwise subsequently bind to the target site and would further aggravate the symptoms or prolong the symptoms already present. Such a regimen appeared feasible as ASV is known to be ineffective against the venom that has already bound to target receptors. All the patients were initially ventilated using A/C mode of ventilation. By employing such an early aggressive approach, we aimed to attain early recovery, improve survival and decrease the duration of mechanical ventilation, and also the incidence of associated complications. Mean total amount of ASV used was 412 ml, which was considerably less than the total amount used in other studies employing a high total dose ASV, but a low initial bolus dose if ASV.[5] This suggests that by employing a high initial bolus dose of 200 ml ASV, we can actually reduce the total amount of ASV consumed until complete neurological recovery. We have also used neostigmine and atropine as these class of drugs have been shown to be effective as a supportive measure in the management of neurotoxic snake bite.[7]

Mean duration of mechanical ventilation on A/C mode in our study was 30.89 hours with 34 patients (58.6%) requiring A/C mode of ventilation for less than or equal to 24 hours. Mean duration of weaning was 7 hours. The total duration of mechanical ventilation in our study was considerably less as compared to other similar studies.[5] These studies have not employed such a high initial bolus dose of 200 ml ASV. This suggests that by employing such a regimen we can possibly reduce the duration of mechanical ventilation and associated complications. Four patients developed ventilator associated pneumonia. Two patients expired, one due to ventilator associated pneumonia and the other due to complications of aspiration pneumonia and septicemia. A reduced total duration of mechanical ventilation resulted in a lower incidence of ventilator associated complications.

By this study we conclude that in the management of neurotoxic snake bite, administration of a high initial bolus dose of 200 ml ASV and repeated doses of 100 ml ASV every 6 hours until signs of neurological recovery, given along with neostigmine and atropine and supported by A/C mode of ventilation resulted in an early recovery, a reduced total dose of ASV consumed, reduced the duration of mechanical ventilation, reduced the incidence of complications and thus was much more cost effective. However, more such studies need to be carried out to formulate a protocol for ASV administration and management of neurotoxic snake bite. Until such a universal protocol has been made, treatment using high initial dose of ASV along with ventilatory support appears to be the most effective.
